# Chronic Myeloid Leukemia Patients in Prolonged Remission following Interferon-α Monotherapy Have Distinct Cytokine and Oligoclonal Lymphocyte Profile

**DOI:** 10.1371/journal.pone.0023022

**Published:** 2011-08-09

**Authors:** Anna Kreutzman, Peter Rohon, Edgar Faber, Karel Indrak, Vesa Juvonen, Veli Kairisto, Jaroslava Voglová, Marjatta Sinisalo, Emília Flochová, Jukka Vakkila, Petteri Arstila, Kimmo Porkka, Satu Mustjoki

**Affiliations:** 1 Hematology Research Unit, Biomedicum Helsinki, Helsinki University Central Hospital (HUCH), Helsinki, Finland; 2 Department of Hemato-Oncology, University Hospital Olomouc, Olomouc, Czech Republic; 3 Department of Clinical Chemistry and TYKSLAB, Turku University Central Hospital, Turku, Finland; 4 2nd Department of Internal Medicine, Clinical Hematology, University Hospital Hradec Králové, Hradec Králové, Czech Republic; 5 Department of Internal Medicine, Tampere University Hospital, Tampere, Finland; 6 Department of Hematology and Transfusiology, University Hospital Martin, Martin, Slovak Republic; 7 Department of Bacteriology and Immunology, Haartman Institute, University of Helsinki, Helsinki, Finland; 8 Division of Hematology, Department of Medicine, Helsinki University Central Hospital (HUCH), Helsinki, Finland; 9 Laboratory of Hematology, Department of Clinical Chemistry, Helsinki University Central Hospital (HUCH), Helsinki, Finland; Rega Institute, University of Leuven, Belgium

## Abstract

Before the era of tyrosine kinase inhibitors (TKIs), interferon-alpha (IFN-α) was the treatment of choice in chronic myeloid leukemia (CML). Curiously, some IFN-α treated patients were able to discontinue therapy without disease progression. The aim of this project was to study the immunomodulatory effects of IFN-α in CML patients in prolonged remission and isolate biological markers predicting response. Due to rarity of patients on IFN-α monotherapy, a relatively small cohort of patients still on treatment (IFN-ON, n = 10, median therapy duration 11.8 years) or had discontinued IFN-α therapy but remained in remission for >2 years (IFN-OFF, n = 9) were studied. The lymphocyte immunophenotype was analyzed with a comprehensive flow cytometry panel and plasma cytokine levels were measured with multiplex bead-based assay. In addition, the clonality status of different lymphocyte subpopulations was analyzed by TCR γ/δ rearrangement assay. Median NK-cell absolute number and proportion from lymphocytes in blood was higher in IFN-OFF patients as compared to IFN-ON patients or controls (0.42, 0.19, 0.21×10^9^/L; 26%, 12%, 11%, respectively, p<0.001). The proportion of CD8+ T-cells was significantly increased in both patient groups and a larger proportion of T-cells expressed CD45RO. Most (95%) patients had significant numbers of oligoclonal lymphocytes characterized by T-cell receptor γ/δ rearrangements. Strikingly, in the majority of patients (79%) a distinct clonal Vγ9 gene rearrangement was observed residing in γδ^+^ T-cell population. Similar unique clonality pattern was not observed in TKI treated CML patients. Plasma eotaxin and MCP-1 cytokines were significantly increased in IFN-OFF patients. Despite the limited number of patients, our data indicates that IFN-α treated CML patients in remission have increased numbers of NK-cells and clonal γδ^+^ T-cells and a unique plasma cytokine profile. These factors may relate to anti-leukemic effects of IFN-α in this specific group of patients and account for prolonged therapy responses even after drug discontinuation.

## Introduction

The Philadelphia chromosome (Ph) resulting from the reciprocal translocation between chromosomes 9 and 22 is the hallmark of chronic myeloid leukemia (CML). The t(9;22) translocation leads to the formation of the *BCR-ABL* oncogene and produces a fusion protein which has an autonomous tyrosine kinase activity [1]. The discovery of tyrosine kinase inhibitors (TKIs) has dramatically improved the survival of CML patients [2], [3], [4]. However, they are not considered to be curative since they do not eliminate all Ph^+^ cells and discontinuation of the therapy often leads to disease relapse [5].

Before the TKI therapy era, interferon alpha (IFN-α) was the treatment of choice in CML [6]. Only a small proportion of patients (10–20%) achieved a complete cytogenetic remission (CCyR), but these patients had a prolonged survival [7], [8]. Recent multicenter studies have shown that combination of IFN-α with the TKI imatinib improves the therapy outcome [9], [10], [11]. Also studies evaluating the successful treatment discontinuation in CML have suggested that IFN-α therapy may improve the possibility to stop TKI therapy [5], [12]. The mechanism of action of IFN-α therapy is incompletely understood; the drug exerts both direct cytostatic and immunomodulatory effects on leukemic cells. It can down-regulate the expression of the *BCR-ABL1* gene, and activate several transcriptional factors that regulate cell proliferation, maturation, and apoptosis [13], [14], [15], [16], [17]. IFN-α can also induce recognition and elimination of CML cells by the immune system [18], [19], [20], [21]. Recent studies have also suggested that it can promote the cycling of normal quiescent hematopoietic stem cells [22]. If similar mechanism of action occurs with dormant leukemic stem cells (LSCs), IFN-α treatment may induce their cycling and thereby expose LSCs to the effects of TKIs and chemotherapeutic agents.

The most striking evidence of the immunomodulatory effects of IFN-α comes from studies which have shown that a significant proportion of IFN-α treated patients in prolonged CCyR were able to discontinue treatment without imminent disease relapse. However, many of these patients still have detectable minimal residual disease [23], [24]. It would be important to understand the mechanisms of drug-induced cure and to assess which factors are important in the maintenance of residual tumor cell dormancy.

The aim of this project was to study the immunomodulatory effects of IFN-α treatment in two unique CML patient populations: (1) patients in prolonged remission during IFN-α monotherapy and (2) patients in prolonged remission after IFN-α monotherapy discontinuation. Such patients are very rare nowadays as TKI therapy has replaced IFN-α in the treatment of CML and therefore, the sample size in this study is limited. However, also with a small number of patients we were able to find distinct changes in the immunoprofile of IFN-α treated patients and these findings should be confirmed in upcoming clinical studies evaluating the effect of IFN-α in the treatment of CML.

## Methods

### Ethics statement

The study was conducted in accordance with the principles of the Helsinki declaration and was approved by the Helsinki University Central Hospital and University Hospital Olomouc Ethics Committees. Written informed consents were obtained from all patients and healthy controls prior to sample collection.

### Study patients and samples

All CML treating physicians in Finland, Czech and Slovak republic were contacted and we were able to identify 19 chronic phase CML patients treated with IFN-α monotherapy. None of the patients were previously treated with TKI therapy. 10 patients were using IFN-α at the time of sampling (age at sampling 33–74 years; median time for treatment 142 months, range 63–231), while 9 patients had discontinued the therapy (age at sampling 45–68 years; median time on treatment 93 months, range 57–132) and stayed in remission at least 2 years (median time without treatment 53 months, range 24–96 months). The reasons for IFN-α discontinuation were request of the patient and/or side effects of the therapy. The majority of the patients have been in CMR for a long period and only in some patients BCR-ABL fusion gene was detectable, but there was the plateau in the transcript level. More detailed patient characteristics are presented in table 1. From 7 patients (4 IFN-ON and 3 IFN-OFF) a follow-up sample (time between samples 15–31 months) was available. As controls we included 4 patients with myeloproliferative neoplasms (MPN) treated with IFN-α (3 patients with essential trombocythemia and one patient with polycythemia vera; age at sampling 26–62 years; median time for therapy 43 months, range 25–108) and 43 healthy volunteers (age at sampling 20–64 years).

**Table 1:** CML patient characteristics (n = 19).

No	Dg	Sex	Age^a^(years)	Sokal score	Disease duration (months)	Course of IFN-α therapy^b^	Response	HLA-A*0201	PR1 %^f^
**1.**	CML CP	M	28	-	358	5 MU/3x weekly (1. year),3 MU/3x weekly – cont.	MCyR	Neg	ND
**2.**	CML CP	F	63	-	63	4.5 MU/daily (1. year),3 MU/3x weekly – cont.	MCyR	Pos	ND
**3.**	CML CP	F	45	-	160	5 MU/daily (1. year),3 MU/3x weekly – cont.	CMR	Neg	ND
**4.**	CML CP	M	62	LR	144	auto-PBSCT^c^,1.5 MU/2x weekly – cont.	MMR	Neg	ND
**5.**	CML CP	F	35	LR	240	2 MU/3x weekly – cont.	CMR	Pos	0.6%
**6.**	CML CP	F	39	-	200	busulfan^d^,1.5 MU/2x weekly – cont.	MMR	ND	ND
**7.**	CML CP	M	29	-	163	4.5 MU/6x weekly – cont.	CMR	Pos	0.9%
**8.**	CML CP	F	21	-	148	mini-ICE, 3 MU/daily – cont.	CMR	Pos	3.9%
**9.**	CML CP	F	55	LR	102	3 MU/daily – cont.	CCyR	ND	ND
**10.**	CML CP	M	58	LR	147	3 MU/daily – cont.	CMR	ND	ND
**11.**	CML CP	M	44	LR	204	IFN-α (10 years),7 years no therapy	CMR	Pos	0.3%
**12.**	CML CP	M	53	LR	170	IFN-α (11 years),3 years no therapy	CMR	Neg	ND
**13.**	CML CP	F	53	LR	134	auto-PBSCT^c^, IFN-α (8 years),3 years no therapy	CMR	Pos	0.6%
**14.**	CML CP	M	59	LR	86	IFN-α (6 years),2 years no therapy	CMR	ND	ND
**15.**	CML CP	M	42	LR	84	CML-8^e^ (5 years),2 years no therapy	CMR	ND	ND
**16.**	CML CP	M	36	LR	112	auto-PBSCT^c^, IFN-α (5 years),4 years no therapy	CMR	ND	ND
**17.**	CML CP	F	41	LR	172	auto-PBSCT^c^, IFN-α (7 years),7 years no therapy	CMR	ND	ND
**18.**	CML CP	F	54	IR	168	IFN-α (9 years),4 years no therapy	CMR	Pos	0.9%
**19.**	CML CP	F	53	IR	152	IFN-α (8 years),4 years no therapy	CMR	ND	ND

No, patient number; dg, diagnosis; CML; chronic myeloid leukemia; CP, chronic phase; auto-PBSCT, autologous peripheral blood stem cell transplantation; F, female; M, male; IFN-α, interferon-α; IR, intermediate risk; LR, low risk; MCyR, major cytogenetic response, CCyR, complete cytogenetic response; CMR, complete molecular response; MMR, major molecular response; cont, continues; HLA, human leukocyte antigen; PR1, a peptide for proteinase-3; ND, not done.

a Age at diagnosis;

b course of therapy; all patients were pretreated with hydroxyurea if not otherwise mentioned;

c auto-PBSCT, priming miniICE, conditioning high-dose busulfan;

d busulfan sequential therapy regimen after pre-treatment with hydroxyurea;

e IFN-α administration in CML-8 protocol (combined with peroral cytarabine at the start);

f percentage of PR-1 specific cells from CD8+ T-cells.

Fresh peripheral blood (PB) samples were collected from all patients and healthy controls. Mononuclear cells (MNCs) were separated by Ficoll gradient centrifugation (GE healthcare, Buckinghamshire, UK).

Blood cell counts were obtained from routine laboratory tests. Molecular genetic analysis of BCR-ABL transcripts was performed with real-time quantitative PCR in quality-controlled laboratories. The cytogenetic response was assessed using conventional G-banding technique.

### Immunophenotyping of PB lymphocytes and leukocyte subpopulations

Immunophenotyping was done with a 6-color flow cytometry panel including antibodies against the following antigens: CD3, CD4, CD8, CD16/56, CD19, CD45, CD57, CD45RA, CD45RO, T-cell receptor (TCR)-α/β, TCR-γ/δ, CCR2, CCR3, and Vγ9 with isotype controls. Regulatory T-cells (Treg) were analyzed with CD3, CD4, CD25, and FOXP3 (clone PCH101, eBioscience, San Diego, CA, USA) antibodies.

From the lymphocyte population, proportions of T-, B-, NKT-like (T-cells with CD16/56 NK-cell marker) and NK-cells were evaluated based on their surface antigen expression (CD3^+^, CD19^+^, CD3^+^CD16/56^+^, CD3^neg^CD16/56^+^, respectively). From the CD3^+^ T-cells, proportions of CD4^+^ helper and CD8^+^ cytotoxic T-cell subpopulations were identified. Also TCR status, CD45RA, and CD45RO cell surface antigens were analyzed from T-cells and CD4^+^ and CD8^+^ subtypes. Absolute numbers of defined cell types were counted from PB cell populations using relative numbers and total white blood cell counts. All antibodies were purchased from BD Biosciences (San Diego, CA, USA) if not otherwise mentioned. Cells were stained according to manufacturer's recommendations. 1–5×10^5^ cells were analyzed with FACSCanto (Beckton Dickinson, San Jose, CA, USA). The data analysis was done with FACSDiva software (version 6.0, BD Biosciences).

### Analysis of HLA-A2 and measurement of PR1 specific T-cells

10 patients (table 1) were analyzed for their HLA-A2 expression with OLERUP SSP™ HLA-kit (Olerup SSP AB, Saltsjobaden, Sweden) for locus A according to the manufacturer's recommendations. Approximately 5 million PB MNC from HLA-A*0201 positive patients were stained with the following antibodies: CD3, CD4, CD8, and PR1 iTAg™ MHC Class I human Tetramer (Beckman Coulter, Brea, CA, USA). 5×10^5^ CD8^+^ cells were analyzed with FACSAria and the data analysis was done with FACSDiva software.

### Fluorescence activated cell sorting (FACS) of αβ^+^ and γδ^+^ T-cells

Approximately 10 million mononuclear cells (MNCs) from 10 CML patients, 4 MPN patients, and 3 healthy volunteers (mean age 45) were stained with antibodies against the following antigens: CD45, CD3, TCR α/β, and TCR γ/δ. Lymphocyte populations were gated and sorted as following: CD3^+^ TCR αβ^+^, and CD3^+^ TCR *γδ* ^+^. All antibodies were purchased from BD and sorting of cells was performed on FACSAria. Purity of the sorted fractions was confirmed with flow cytometry to be close to 100%.

### DNA extraction

Genomic DNA was isolated from frozen, or sorted PB MNCs by Genomic DNA from Tissue; NucleoSpin® Tissue or Tissue XS (Machery-Nagel, Düren, Germany) according to manufacturer's instruction. DNA concentration and purity was measured with NanoDrop (Thermo Scientific, Waltham, MA, USA). DNA was stored at −20°C.

### Detection and sequencing of TCR γ- and δ-gene rearrangements by PCR

TCR γ- and δ-gene rearrangements were studied by PCR analysis using 12 primer pairs for γ-gene and 6 primer pairs for δ-genes detecting most of the known TCR γ- and δ-gene rearrangements. The lower detection limit of the assay is 1–5% of clonal cells among polyclonal lymphocytes. The analysis was done according to the BIOMED-1 PCR protocol [25]. Clonal products were identified by heteroduplex analysis, sequenced and analyzed as described previously [26].

### Design of individual allele-specific oligonucleotide (ASO) primers and establishment of clones in different cell populations with RQ-PCR

Patient-specific allele-specific oligonucleotide (ASO) primers for real-time quantitative PCR (RQ-PCR) were designed as explained previously [26]. Basically, the ASO primers were designed to match the hypervariable junction regions of the sequenced TCR δ- and TCR γ-gene rearrangements. TaqMan RQ-PCR was performed using the ASO-primers together with a consensus primer and a TaqMan probe, which were selected according to the D- and J-gene present in the detected clonal rearrangement (primer and probe sequences available from the authors upon request). DNA from sorted cell populations or diagnostic phase (patient 18, table 1) was amplified in duplicate reactions using TaqMan Universal PCR Mastermix (Applied Biosystems).

### Luminex assay of cytokines

The plasma of 10 healthy volunteers and patients treated with IFN-α, including 19 CML patients and 4 control MPN patients, were analyzed for following cytokines; interleukin-1b (IL-1b), IL-1RA, IL-2, IL-2R, IL-4, IL-5, IL-6, IL-7, IL-8, IL-10, IL-12, IL-13, IL-15, IL-17, TNF-α (tumor necrosis factor alpha), IFN-α (interferon alpha), GM-CSF (granulocyte macrophage colony-stimulating factor), MIP-1a (macrophage inflammatory protein 1a /CCL3), MIP-1b (macrophage inflammatory protein 1b /CCL4), IP-10 (IFN-inducible protein 10 /CXCL10), MIG (monokine induced by interferon gamma /CXCL9), Eotaxin (CCL11), Rantes (CCL5), MCP-1 (monocyte chemoattractant protein 1 /CCL2), and IFN-γ (interferon gamma) using a Human Cytokine 25-plex Panel (Invitrogen) accoring to the manufactorer's instructions. Measurement and analysis was performed in Bio-Plex™ 200 System (BioRad).

### Statistical analysis

Statistics of the immunophenotyping results were done with SPSS version 16.0. Assessment of the statistical significance of the other experiments was done with GraphPrism (GraphPad Software Inc., CA, USA). Cytokine profiles were analyzed with nonparametric Mann-Whitney test and t test. P<0.05 was considered statistically significant.

## Results

### Patient characteristics

All CML patients studied were in chronic phase. None of the patients were treated with TKI therapy, or had undergone allogeneic stem cell transplantation making this group of patients rare and therefore limited. The median time on IFN-α therapy was 11.8 years (range 5.3–19.3 years) in patients who were still on treatment (IFN-ON) and 7.8 years (4.8–10.8) in patients who had discontinued therapy (IFN-OFF, table 1). In IFN-OFF patients, the median time without treatment before blood sampling was 4.4 years (2.0–8.0). Most (8/10) IFN-ON patients had at least CCyR and 5 were in CMR. 2 patients had major cytogenetic response, but the disease was stable and the treatment had continued for more than 5 years in both patients. The IFN-α dose differed markedly between patients ranging from 1.5 million units (MU) twice weekly to 3 MU daily (table 1) mirroring well the real clinical situation as the tolerated and effective dose of IFN-α varies greatly between individual patients. 85% of patients (11 of 13) from whom data was available belonged to low Sokal risk group and 15% to intermediate risk group.

### Increased amount of NK-cells in patients who have successfully discontinued IFN-α therapy

**Figure 1:**
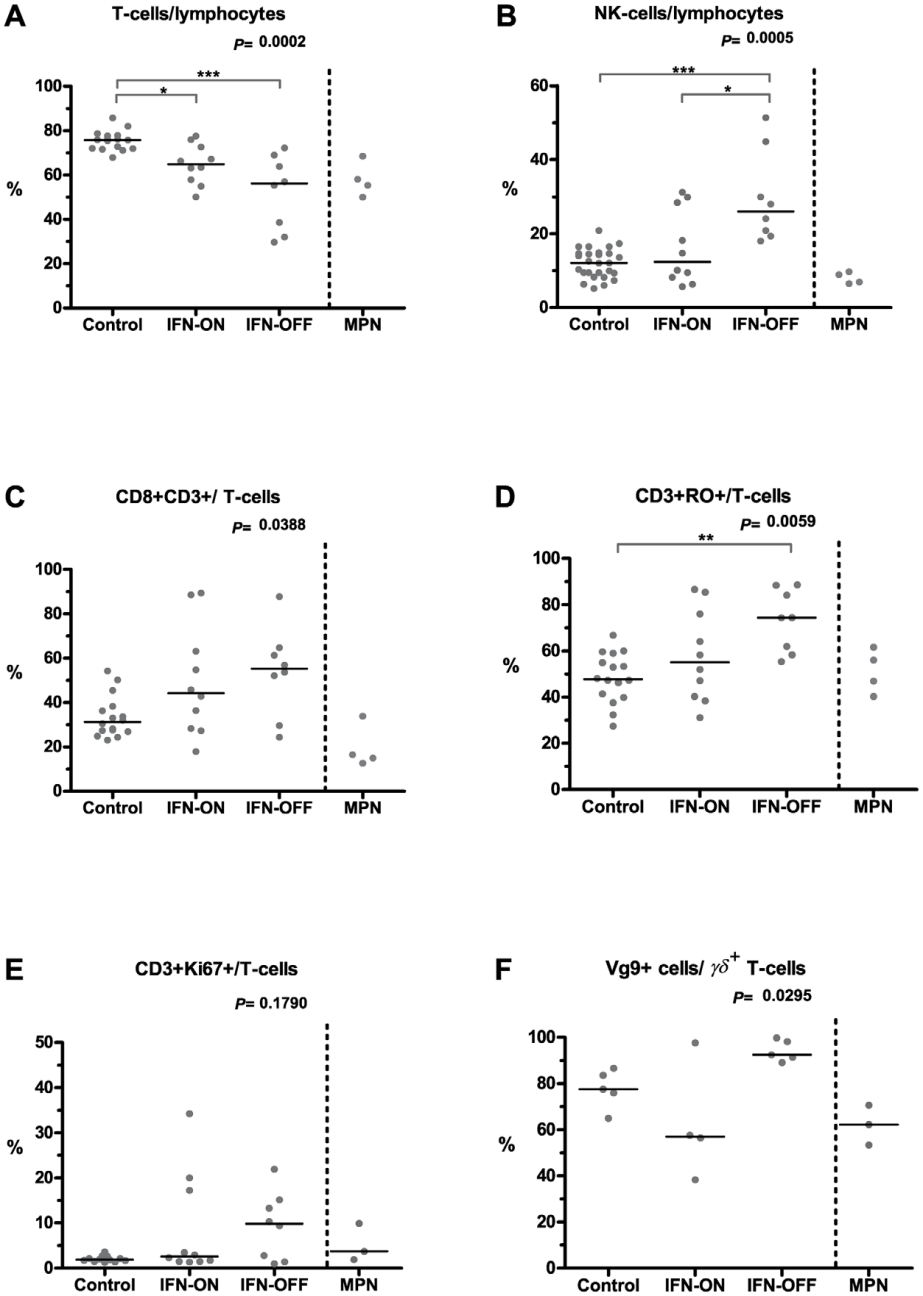
Flow cytometry analysis of basic lymphocyte subpopulations. Immunophenotyping was done with 6-color flow cytometry from healthy controls (n = 16), IFN-α-treated CML patients (IFN-ON, n = 10), and CML patients who had discontinued IFN-α therapy (IFN-OFF, n = 9). Figures represent relative proportions of analyzed cells and corresponding absolute numbers can be found in Table S1. Statistical significance of differences in continuous variables was assessed by a non-parametric analysis of variance using the Kruskal-Wallis test (P-values reported in figures) and in case of a significant main effect, pairwise comparisons of patient groups were calculated using Dunn's multiple comparison test (statistically significant differences between groups are marked with asterisks and line). For the analysis of Vγ9^+^ cells (panel F), samples from 5 healthy controls, 4 IFN-ON, and 5 IFN-OFF patients were available. The figures also include results from four patients with myeloproliferative neoplasm (MPN), but they were not included in the statistical analysis.

Immunophenotyping of basic leukocyte subpopulations was done with a multicolor flow cytometry panel. Median total lymphocyte count did not differ significantly between healthy controls (1.81×10^9^/l) and IFN-ON (1.52×10^9^/l) or IFN-OFF patients (1.52×10^9^/l). The proportion of CD3^+^ T-cells from total lymphocyte population was decreased in both CML patient groups when compared to healthy volunteers (67% in IFN-ON, 56% in IFN-OFF vs. 73% in healthy controls, p = 0.0002)(Figure 1A, Table S1). Similarly, the absolute T-cell numbers were lower in the patient groups (0.89×10^9^/l, 0.82×10^9^/l vs. 1.3×10^9^/l, respectively, p = 0.0049) as well as the amount of NKT-like cells (Table S1).

The proportion of NK-cells from total lymphocyte population was significantly increased in the IFN-OFF group (26%) as compared to IFN-ON group (12%) or to healthy controls (11%) (Figure 1B, p = 0.0005). Absolute NK-numbers differed similarly with marked variation in the IFN-ON group of patients (Table S1).

The CD4/CD8 ratio was lower in IFN-ON (1.3) and IFN-OFF (0.8) groups in comparison to healthy volunteers (2.2, p = 0.0402). This predominantly reflected the higher proportion of cytotoxic CD8^+^ T-cells (44% of total T-cell population in IFN-ON, 55% in IFN-OFF vs. 31% in healthy controls, p = 0.0388, figure 1C, Table S1). Furthermore, the absolute CD4+ T-cell numbers were decreased both in IFN-ON (0.47×10^9^/l) and IFN-OFF (0.33×10^9^/l) groups when compared to healthy controls (0.84×10^9^/l, p = 0.0010, Table S1). IFN-α treated MPN patients had comparable numbers of NK-cells and CD8+ T-cells as healthy controls (figure 1 B, C).

There was no difference in the proportion or absolute counts of B-cells (Table S1). No significant difference was observed in the T-cell receptor (TCR) subtype distribution between healthy volunteers and the IFN-α subgroups (Table S1).

### CML patients treated with IFN-α have a higher proportion of CD3^+^ CD45RO^+^ T-cells

We further examined the memory and activation status of T-cells. Interestingly, both CML patient groups had significantly larger proportion of CD45RO-positive T-cells, which are antigen encountered memory cells (58% in IFN-ON group and 74% in IFN-OFF group vs. 44% in healthy volunteers, p = 0.0059)(figure 1D, Table S1). No significant difference was observed in the proportion of CD57 activation antigen expressing T-cells (Table S1), but patients in the IFN-OFF group in particular had proliferating CD3+ T-cells as assessed by Ki-67 antigen expression (figure 1E).

### Increased proportion of regulatory T-cells in IFN-α treated CML patients

The median proportion of Tregs from CD4^+^ lymphocytes was significantly increased in IFN-OFF subgroup (6.1%) compared to healthy volunteers (3.8%) and IFN-ON subgroup (5.2%) (p = 0.0114, Table S1). However, no difference was observed in absolute Treg numbers (Table S1).

### Immunoprofile stays stable in follow-up samples

From 7 patients (4 IFN-ON and 3 IFN-OFF) a follow-up sample was available. The time between the first and second sample varied from 15 to 31 months. The proportion of NK-cells was stable over time and no significant variation was detected in the values between 2 time-points (data not shown). Similarly, in other cell types (CD8+ cells and CD45RO+ cells) in which significant differences were observed between the study cohorts, the results between 2 time-points did not differ markedly.

### HLA-A2 and PR1 specific T-cells

To be able to analyze the proportion of PR1 specific T-cells, patients' HLA-A2 status was determined as PR1 MHC Class I human tetramer antibody staining is restricted only to HLA-A2 positive patients. Suitable samples for HLA-A2 analysis were available from 10 CML patients, and 7 were positive for HLA-A*0201 (table 1). All these 7 patients had detectable PR1 specific CD8+ T-cells with the proportion varying between 0.3% and 3.9% of total CD8^+^ cell population (table 1).

### Clonal lymphocytes are common in IFN-α treated patients

As our previous studies have shown that the majority of CML patients have clonal lymphocytes already at the time of diagnosis and that they expand during dasatinib (2^nd^ generation TKI) therapy [26], we wanted to study the presence of clonal lymphocytes in IFN-α treated patients. Clonality of the cells was determined by using a sensitive TCR γ/δ rearrangement PCR assay (sensitivity of the assay is 1% of clonal cells within the total assayed cell population). 22 healthy volunteers were analyzed as controls. In three healthy volunteers (14%), a clonal TCR γ-rearrangement was confirmed. However, no clonal TCR δ-gene rearrangements were detected in healthy controls (table 2). The healthy individuals with clonal T-cells (male 44 years, male 33 years, and female 47 years) were asymptomatic and had normal blood counts.

**Table 2:** Sequence confirmed clonal TCR γ- and δ-gene rearrangements.

No.	Patient group	TCR delta primer pairs						TCR gamma primer pairs											
		1	2	3	4	5	6	1	2	3	4	5	6	7	8	9	10	11	12
**healthy**	-											***X***							
**healthy**	-										X								
**healthy**	-													X					
**1**	IFN-ON		X	X							X	***X***		X					
**2**	IFN-ON		X	X								***X***						X	
**3**	IFN-ON																		
**4**	IFN-ON		X	X				X	X		X	***X***					X		
**5**	IFN-ON		X									***X***							
**6**	IFN-ON											***X***							
**7**	IFN-ON													X			X		
**8**	IFN-ON			X															
**9**	IFN-ON			X								***X***					X		
**10**	IFN-ON		X									***X***					X	X	
**11**	IFN-OFF	X		X				X	X			***X***							X
**12**	IFN-OFF	X	X	X							X	***X***							
**13**	IFN-OFF			X					X		X								
**14**	IFN-OFF			X								***X***							
**15**	IFN-OFF			X								***X***							
**16**	IFN-OFF								X										
**17**	IFN-OFF										X	***X***							
**18**	IFN-OFF											***X***		X					
**19**	IFN-OFF		X	X								***X***							
**20**	MPN																		
**21**	MPN																		
**22**	MPN	X		X							X	***X***							
**23**	MPN	X		X							X	***X***		X					

No refers to patient number in the Table 1; IFN-ON, CML patients with ongoing IFN-α therapy; IFN-OFF, CML patients who have discontinued the therapy; MPN myeloproliferative neoplasm.

Clonality was determined by PCR and gel analysis using 12 primer pairs for the TCR γ-gene rearrangements and 6 primer pairs for TCR δ-gene rearrangements. Positive clonal PCR products were confirmed with sequencing and are marked in the columns as X. Clonal TRGV9*01 / TRGJP*01 rearrangements are marked with bold and italic.

In contrast, 18 of 19 (95%) CML patients treated with IFN-α had a clonal rearrangement in TCR γ- and/or δ-genes (table 2). Both IFN-ON and IFN-OFF patients had frequently clonal TCR γ- (80% vs. 100%, respectively) and TCR δ-gene rearrangements (60% vs. 67%). In most patients more than one clonal TCR rearrangement was confirmed (15 of 19; 79%). 2 of 4 (50%) MPN patients included in this study had clonal TCR γ- and TCR δ-gene rearrangements. In one CML patient and two MPN patients (male 34 years and female 26 years) no clonal rearrangements could be detected with the set of primers used in this study.

Two CML patients had a diagnostic phase sample available (patients 9 and 18). Patient 18 showed no clonal lymphocytes at diagnosis, while patient 9 had one clonal product detected with primer pair TCR gamma 10 (table 2). A clone could be detected with the same pair 8 years later during IFN-α therapy, but the junction region sequence varied slightly suggesting that the clone was not identical or it has evolved during the years.

### A unique pattern of TCR γ/δ rearrangements is observed in IFN-α treated patients

Surprisingly, in the majority of CML patients either on IFN-α therapy (70%) or who had been able to discontinue the therapy (78%), a clonal rearrangement was detected with the same primer pair Vg2-Jg1.2 (table 2, TCR γ primer pair 5). Also 2/4 MPN control patients had a clone detected with this pair of primers. However, no similarities could be found in the specific junction regions (Table S2).

A similar finding was observed in TCR δ-gene rearrangements detected with primer pairs 1 and 3 (Vδ1-Jδ1 and Vδ2-Jδ1, both generating complete rearrangements, table 2). 9 of 14 CML patients (64%) who had a rearrangement detected with Vg2-Jg1.2 had also a clone detected with Vδ1-Jδ1 and/or Vδ2-Jδ1 (table 2). Also the two MPN patients had the both TCR genes rearranged. Similarly, no pattern could be found in the specific junction regions (Table S3).

The same unique pattern has not been observed earlier in TKI-treated patients [26] and was not either seen in healthy volunteers (1 positive finding in 47 years old female of total 22 volunteers studied).

### Clonal TCR γ- and δ-gene rearrangements are found in the γδ^+^ T-cell population

**Figure 2:**
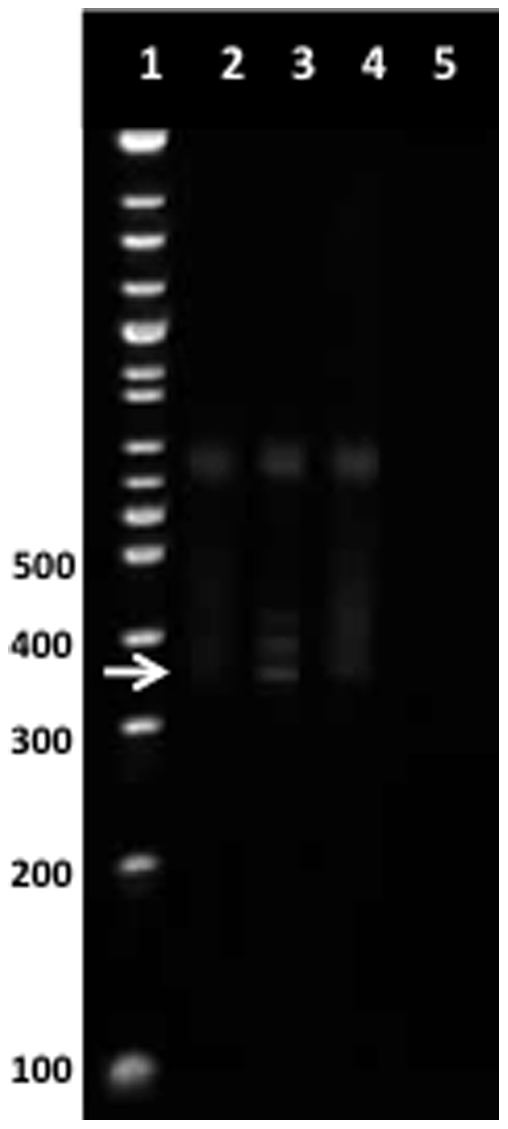
Clonal Vγ9 rearrangement in γδ^+^ T-cells. Example of Vδ2Jγ9 clonality detection in patient number 4 (Table 1). PCR with Vg2-Jg1.2 primers was carried out on FACS sorted cell fractions and the clonal products were identified by heteroduplex analysis. Lane 1, DNA ladder; lane 2 αβ^+^ T-cell fraction; lane 3 γδ^+^ T-cell fraction; lane 4 pool of healthy controls showing polyclonal smear; lane 5, water control. Clonal PCR product in the γδ^+^ T-cell fraction is shown with a white arrow.

To further elucidate in which cell population the clonal cells resided, PB MNC from 8 CML and 2 MPN control patients (Table S2 and S3) were sorted with FACS into pure CD3^+^ γδ^+^, and CD3^+^ αβ^+^ T-cell populations. For 3 patients, patient specific (ASO)-primers were designed for the detection of clonal TCR γ- and δ-gene rearrangements (Table S2 and S3) and for the rest of the patients (n = 5) above described PCR protocol was used to detect the site of the clonal population. In all CML and MPN patients, a clonal TCR γ/δ rearrangement was observed within CD3^+^ γδ^+^ T-cell population. Example of one patient is shown in figure 2. αβ+ T-cell populations were negative with corresponding primers.

To be able to see if these specific clonal TCR γ- and δ-gene rearrangements were present in small quantities also in the γδ^+^ T-cell population in healthy controls, we enriched γδ^+^ T-cells from 3 healthy volunteers by FACS. One healthy control showed a clonal population. Of note, the clone was only detected in the purified γδ^+^ T-cell population; PCR done from MNC showed no clonal product indicating that the amount of γδ^+^ T-cell clone is less than 1% from the total MNC. In contrast, in CML patients the clone was detected also in the whole MNC population. However, most of the clones (confirmed by sequencing) were detected against a strong polyclonal background, indicating that majority of γδ^+^ T-cells are polyclonal.

### Increased amount of Vγ9^+^ cells in patients who had discontinued IFN-α therapy

As the majority of IFN-α treated patients had a distinct clonal rearrangement detected with the same primer pair Vg2-Jg1.2 in the γδ^+^ T-cell populations and as it has previously been described that this primer pair recognizes Vδ2Jγ9 T-cells [27], we wanted to analyze the proportion of Vγ9^+^ cells in CML (n = 9) and MPN (n = 3) patients and in healthy controls (n = 5). The majority of γδ^+^ T-cells both in patients and in healthy controls were Vγ9^+^ cells (median percentages for healthy 78%, IFN-ON 57%, and MPN patients treated with IFN-α 62%)(figure 1F). Especially in patients who had discontinued the therapy, nearly all (median percentage 92%) γδ^+^ T-cells were Vγ9^+^ cells (figure 1F).

### CML patients treated with IFN-α have a distinct cytokine profile compared to healthy volunteers

**Figure 3:**
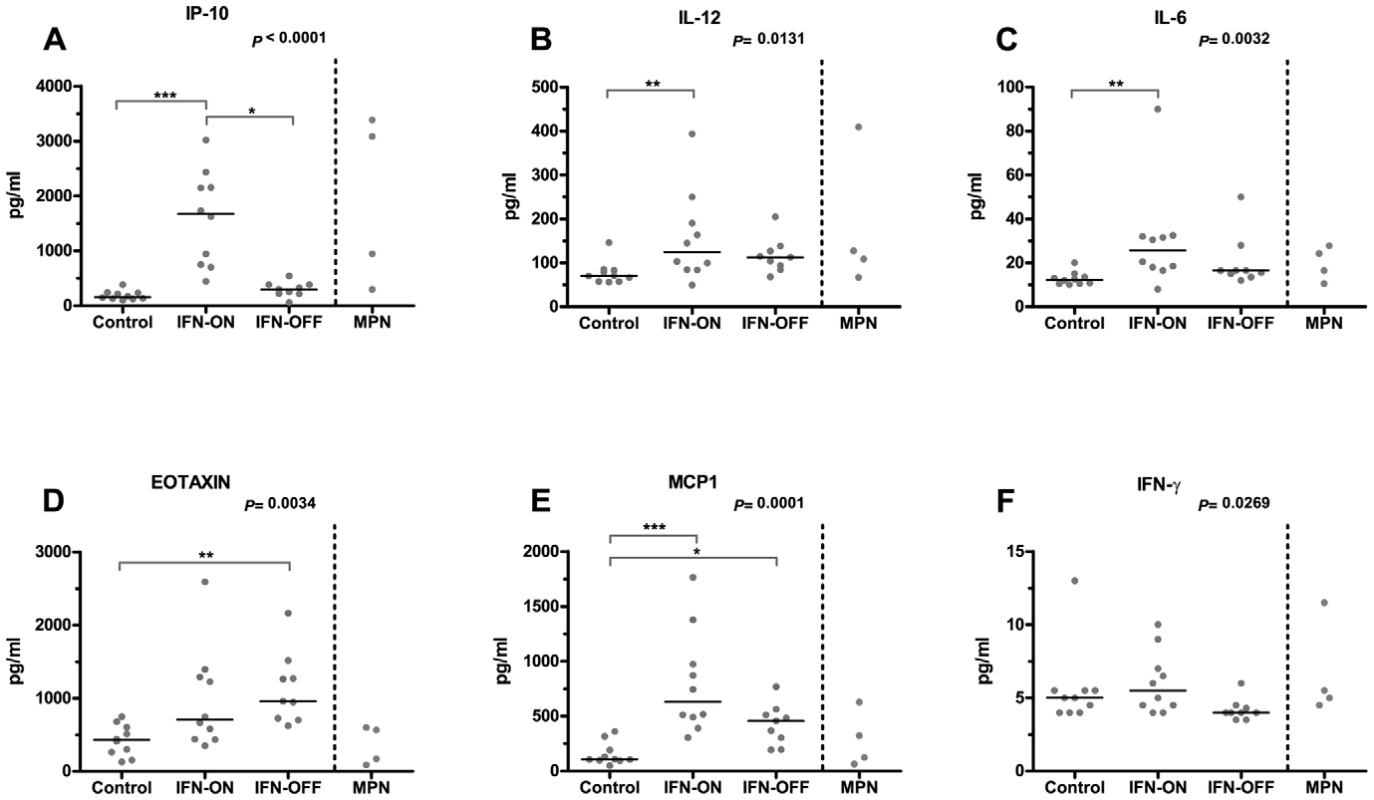
Plasma IP-10, IL-6, IL-12, eotaxin, MCP-1, and IFN-γ cytokine levels. Plasma levels of 25 cytokines were measured with a multiplex bead-based cytokine assay (Luminex®) from healthy controls (n = 10), IFN-α-treated CML patients (IFN-ON, n = 10), and CML patients who had discontinued IFN-α therapy (IFN-OFF, n = 9). Statistical significance of differences in continuous variables was assessed by a non-parametric analysis of variance using the Kruskal-Wallis test (P-values reported in figures) and in case of a significant main effect, pairwise comparisons of patient groups were calculated using Dunn's multiple comparison test (statistically significant differences between groups are marked with asterisks and line). The figures also include results from four patients with myeloproliferative neoplasm (MPN), but they were not included in the statistical analysis.

Plasma cytokine levels were analyzed with a multiplex bead-based cytokine assay assay (Luminex®) measuring 25 different cytokines. IP-10 (CXCL10), IL-6, IL-12, and MCP-1 (CCL2) levels were significantly increased IFN-ON patients when compared with healthy controls (figure 3). As it is known that IFN-α therapy can induce most of these factors [28], it was interesting to observe that also patients who had discontinued IFN-α therapy over 2 years prior to sample collection had higher IL-6, IL-12, IP-10, eotaxin (CCL11), and MCP-1 levels when compared to healthy controls (comparisons between 2 groups were done with Mann-Whitney U-test). Especially plasma eotaxin levels were markedly increased in IFN-OFF group compared to healthy volunteers (1173 and 427.7 pg/ml, respectively; p<0.0001). However, no differences in eosinophil counts were detected (data not shown). Similarly, MCP-1 levels differed markedly between these 2 groups (631.5 vs. 106.5 pg/ml, p = 0.0003) but no differences in monocyte counts were observed (data not shown).

In 4 MPN control patients treated with IFN-α, plasma eotaxin and MCP-1 levels were at the same range as in healthy volunteers (figure 3D–E).

### T-cells from IFN-α treated patients express cytokine receptors for eotaxin and MCP-1

To examine if γδ^+^ and αβ^+^ T-cells have corresponding receptors (CCR2 and CCR3) for the cytokines (MCP-1 and eotaxin, respectively) which were increased in both CML groups, samples from IFN-ON (n = 4), IFN-OFF (n = 3), MPN control patients (n = 3), and healthy volunteers (n = 5) were analyzed with flow cytometry. Both T-cell subsets in all study groups expressed these receptors, although the proportion of CCR2 and CCR3 positive cells were low (0.1%–8.0% of γδ^+^ and αβ^+^ T-cells).

### Possible factors predicting persistent leukemia dormancy after IFN-α discontinuation

**Figure 4:**
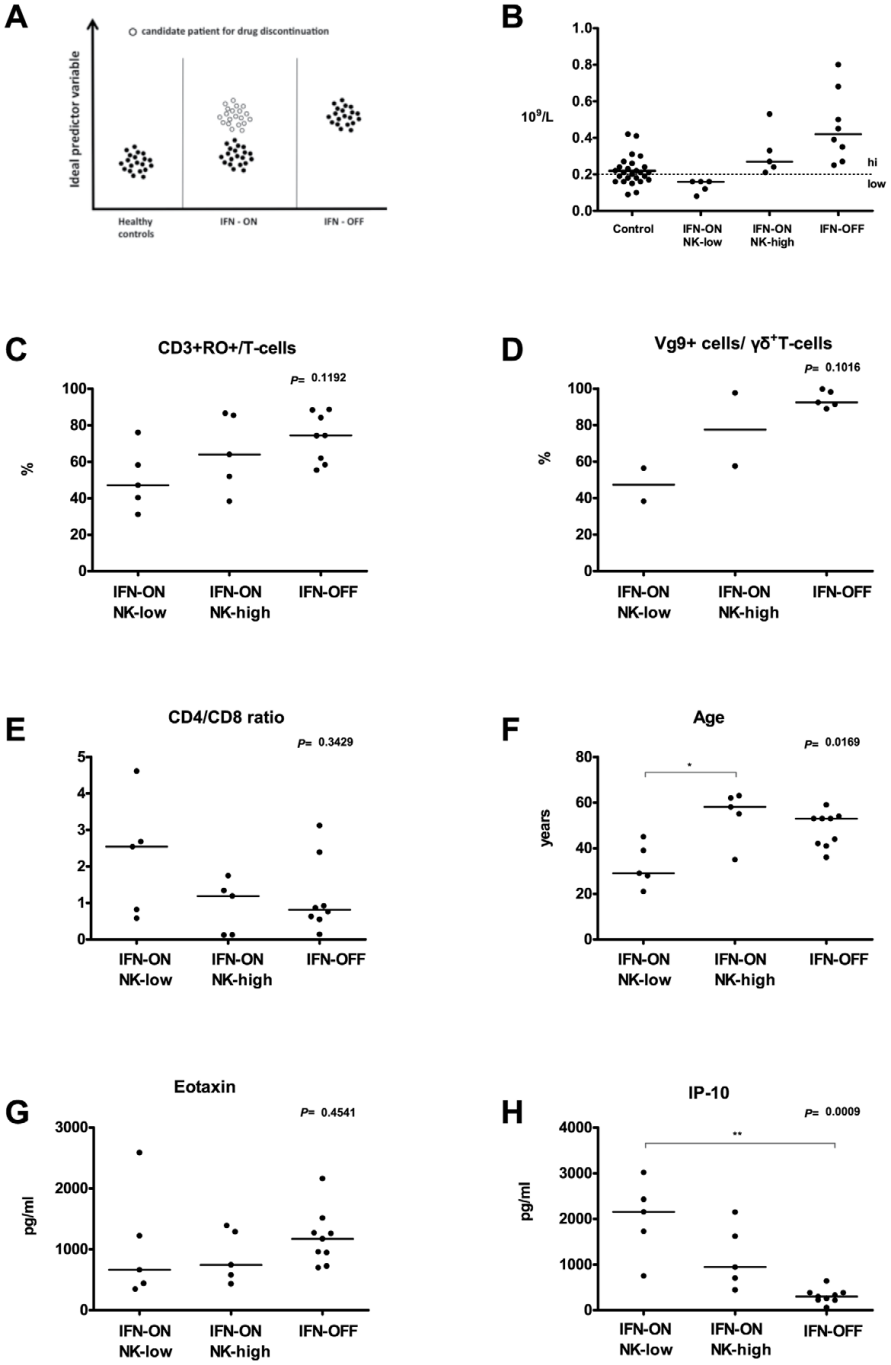
Characteristics of a predictor variable for IFN-α discontinuation. A. Example of a hypothetical variable differentiating patients who can stop IFN-α therapy (empty circle) from those who cannot in the IFN-ON group. B. NK-cell count as a potential predictor variable. The figure presents absolute NK-cell numbers in different patient groups. Patients still using IFN-α therapy were divided in 2 groups based if their absolute NK-cell count was above (IFN-ON NK-high) or below (IFN-OFF NK-low) 0.2×10^9^/l. C–H. Biomarker profile of NK-high and NK-low groups. Patients using IFN-α therapy (IFN-ON) were divided in 2 groups based on the absolute NK-cell count in the peripheral blood. In NK-high group, the absolute NK-cell count was above 0.2×10^9^/l and in NK-low group below the given limit. Other variables are presented based on the group division by NK-cell number and IFN discontinued patients as a separate cohort (IFN-OFF).

Based on previous studies in patients with prolonged complete remission on IFN-α therapy [23], [24], we estimated that approximately 50% of our IFN-ON patients could discontinue IFN-α without imminent disease relapse. Therefore, we were interested to see if it would be possible to find markers that could be used in predicting which patients will relapse after IFN-α discontinuation and which will remain in remission and in the state of leukemia dormancy. A hypothetical variable, which would predict in the IFN-ON group which patients can stop IFN-α, would need to fulfill at least the following criteria: (1) IFN-ON median values of the variable should be significantly different (higher or lower) from IFN-OFF values and (2) IFN-ON values of the variable should have approximately twice the variance of IFN-OFF values (figure 4A).

After review of all the immunological variables analyzed in the present study, both NK cell proportion from lymphocytes and absolute NK-cell count best fulfilled the criteria for a predictor variable for drug discontinuation. To characterize the IFN-ON cohort in more detail we next divided the IFN-ON cohort into two subgroups based on the median NK-cell count of 0.2×10^9^/L: the high NK group (NK-cell count>0.2) constituting patients most likely to remain in remission after IFN-α discontinuation, and the low NK group (<0.2) (figure 4B).

There were no evident differences in the high or low NK groups with regards to gender, duration of disease or IFN-α dose used. A trend for older age and a higher proportion of Vγ9^+^ γδ^+^ and RO^+^ T-cells and lower CD4/CD8 ratio was observed in the IFN-ON NK high and INF-OFF groups as compared to IFN-ON NK low group (figure 4C–F). Clonal γδ^+^ T-cells were more often observed in the IFN-ON NK high (100%) and INF-OFF (75%) groups as compared to IFN-ON NK low group (40%). Interestingly, IP-10 levels were lower in the IFN-OFF as compared to the IFN-ON NK low group with IFN-ON NK high patients showing values in between (figure 4H). No differences in plasma eotaxin levels were observed (figure 4G). However, the number of patients is small and confirmatory studies with larger patient populations are warranted.

## Discussion

CML is considered to be one of the most susceptible malignancies to immune manipulation. Results from allogeneic hematopoietic stem cell transplantation and donor lymphocyte infusion therapies have shown remarkable, curative anti-leukemia effects mediated by alloreactive cytotoxic lymphocytes [29]. Encouraging data from clinical trials with BCR-ABL1 peptide vaccines confirm the clinical usefulness of provoking anti-CML immune responses [30], [31]. Several groups have recently reported a favorable outcome for CML patients treated with imatinib in combination with IFN-α, compared to patients on imatinib monotherapy [9], [10], [11] Furthermore, IFN-α therapy may increase the likelihood to stop treatment without relapse [5], [12]. Most patients who have successfully discontinued the therapy, still have detectable minimal residual disease, but the disease is stable and does not progress [23], [24]. This implies that IFN-α therapy has induced an immune response with currently unknown mechanisms, which is able to keep tumor cells dormant.

In this study, we focused on determining the immunomodulatory effects of IFN-α in patients who had used the drug as monotherapy with an excellent treatment response. As TKIs currently have substituted IFN-α as a first line therapy, this group of patients is quite unique. The heterogeneity of the studied patient population considering IFN-α dose and treatment duration as well as reasons why patients have stopped the treatment needs to be taken into account when evaluating these results, but they also represent well the real clinical situation. The tolerated and effective dose of IFN-α varies greatly between individual patients. However, a common denominator in our patient cohort is the long lasting response to therapy, which can usually be achieved only in a small minority of IFN-α treated patients. The IFN-α treatment duration (median 11.8 years in IFN-ON group and 7.8 years in IFN-OFF group) is also considerably long as well as time without any treatment in IFN-OFF group (4.4 years) and therefore it is unlikely that the possible earlier autologous transplantation plays a major role in this setting.

Despite these limitations in the patient number, we were able to recognize distinct factors correlated with prolonged IFN-α therapy response even after discontinuation of the drug. One of these factors was a shift in the lymphocyte profile in IFN-α treated patients into a more cytotoxic direction. Significant increase of CD8^+^ T-cells was observed in CML patients who were still on IFN-α therapy. In patients, who had been able to discontinue therapy, the NK-cell counts were significantly higher. From a proportion of patients we were able to analyze follow-up samples taken >12 months interval from the first sample and importantly, there was not significant intra-individual variation in immunoprofile between different time points. Both CD8^+^ T-cells and NK-cells display cytotoxic effects on leukemia cells, which may play a role in tumor surveillance. In CML, CD8^+^ T-cells have been reported to recognize leukemic cells [32]. Furthermore, NK-cells have been shown to be able to kill CD34^+^ CML stem cells, which also may play a role in the graft vs. leukemia effect after allogeneic transplantation [33],[34]. Of note, in our previous studies with TKI treated patients, no increase of NK- or CD8^+^ T-cells was observed in imatinib treated patients [35]. Interestingly though, a proportion of dasatinib treated patients developed expansion of CD8^+^ T-cells and/or NK-cells [35], [36], which was associated with superior therapy responses [36].

A number of previous studies have implicated that PR1 (a peptide for proteinase-3) is an important tumor antigen for CTL immune response against CML and that specific anti-PR1 T-cells are involved in the elimination of CML cells [37], [38]. In addition, it has been suggested that induction of a PR1-specific CTL response by IFN-α may contribute to improved molecular response in patients treated with imatinib+IFN-α, and that these patients could discontinue imatinib and continue IFN-α without relapse [12]. PR-1 specific CTLs have also been reported to persist in patients who do not relapse after IFN-α withdrawal [39]. In concord, patients in our cohort also had PR1 specific T-cells detectable in small quantities. No clear differences were found in their amount between patients who were still on IFN-α treatment or who had discontinued the therapy, but this could be also due to small patient number studied.

As our previous report showed that oligoclonal, Ph-chromosome negative lymphocytes are common in CML patients at diagnosis and during TKI therapy, we hypothesized that these cells could also be found in IFN-α treated patients. Indeed, clonal lymphocytes were observed in most (95%) IFN-α treated CML patients. Strikingly, and in contrast to TKI-treated patients or healthy controls, a unique rearrangement pattern was observed in IFN-α treated CML patients. A total of 79% of the patients had a clonal rearrangement detected with the same primer pair (Vg2-Jg1.2) whereas during TKI therapy only 10–20% of the patients displayed similar rearrangement [26] suggesting that this phenomenon was associated with IFN-α therapy. It is however uncertain whether this similar rearrangement pattern could be a consequence of same antigen recognition, as no similarities could be found in the specific junction region. Interestingly though, further characterization of the cell populations revealed that the clonal cells resided only in γδ^+^ T-cell population. It is believed that γδ T-cell responses are driven more by imbalance in the host (e.g. cell transformation and inflammation) than by specific pathogen challenges, which could explain that there were no similarities in the junction regions. One hypothesis propose that these cells can directly recognize antigens in the tissue, including “stress antigens” that are markers of cell infection or transformation [40]. The role of γδ^+^ T-cells in tumor immunology has been disputable, and there are only few reports of their importance in hematology [41]. In myeloma patients, activated Vγ9Vδ2 cells were able to kill malignant plasma cells [42]. Also in acute leukemia, it has been reported that Vγ9Vδ2 cells are increased, and that they are related to better disease-free survival after bone marrow transplantation [42]. In addition, one recent report in CML showed that ex vivo expanded γδ^+^ T-cells were able to kill K562 cells [43]. Our results show for the first time that significant amounts of clonal γδ^+^ T-cells exist in CML patients who can be considered to be cured (patients without the treatment) which suggests that these cells possess protective, anti-leukemic properties *in vivo* in patients.

Cytokines are important mediators of immune cell signaling and chemokines (chemotactic cytokines) induce directed chemotaxis in nearby responsive cells. In our patient cohort we noticed that levels of several cytokines and chemokines (IL-6, IL-12, IP-10, eotaxin, MCP-1, and IFN-γ) were increased in CML patients treated with IFN-α. IFN-α therapy itself may increase plasma concentration of some cytokines, such as IP-10 [28], but interestingly, it remained at higher level in the IFN-α discontinued subgroup as well. Saudemont et al have earlier shown that IP-10 provides protection against leukemia in mice. Mice vaccinated or treated with IP-10 transduced cell lines survived leukemia significantly better than controls, which was explained by increased percentage of NK cells, activation of NK cells and improved cytotoxicity of these cells [44]. These results are in accordance with our results and IP-10 could provide an additional mechanism of elimination of dormant tumor cells [44].

Especially eotaxin and MCP-1 levels were higher in the IFN-α discontinued subgroup as compared to healthy volunteers. The same trend was not seen in the control MPN patients included in this study or in CML patients treated with dasatinib or imatinib (A. Kreutzman, unpublished observations). Increased plasma levels of MCP-1 (CCL2) signify the activation of Th1 type immune response, whereas the presence of eotaxin (CCL11) is an indication of Th2 type response. In line with this dichotomy, patients continuing IFN-α therapy tend to have higher MCP-1 levels than patients who had discontinued therapy. Furthermore, patients who had discontinued the therapy had higher eotaxin levels, which may indicate a switch to a Th2 type response. Both of these cytokines have been related to outcome of cancer patients. Eosinophils secrete eotaxin in response to tumors [45] and high MCP-1 levels are associated with a more favorable prognosis in pancreatic cancer [46]. Our results also confirmed that γδ^+^ T-cells in IFN-α treated CML patients expressed corresponding cytokine receptors (CCR2/MCP-1 and CCR3/eotaxin).

There is a growing interest to find biomarkers predicting which patients could stop IFN-α or TKI therapy without disease relapse. A recent case report suggested that patients who were able to discontinue IFN-α treatment, had an increased amount of CD8^+^CD45RO^+^ memory cells compared to patients who relapsed after imatinib or IFN-α discontinuation [47]. Accordingly, in our limited material, we observed elevated numbers of CD45RO^+^ CD3^+^ T-cells especially in the IFN-α discontinuation subgroup. Similarly, in dasatinib treated CML patients we have earlier reported that majority of CD8^+^ T-cells are CD45RO^+^ [35] and this phenotype is related to better therapy outcome [26], [36]. Mahon et al has recently reported that patients who discontinued imatinib treatment without disease relapse belonged more often to Sokal low risk group and had increased numbers of NK-cells at the time of discontinuation (Mahon FX et al. Blood 2009 114: Abstract 859). Similarly, in our IFN-α cohort, 85% of the patients had low Sokal risk, and absolute and relative NK-cell numbers seem to divide patients into 2 different groups. All patients who had been able to discontinue the therapy had NK-cell counts above 0.2×10^9^/L, whereas approximately half of the patients who still used IFN-α therapy had values less than 0.2×10^9^/L. Interestingly, patients in NK-high group also had more often RO+ T-cells and clonal Vg9 cells thus further refining the predictive immunoprofile. The biomarker profile described in this paper could be a candidate profile when considering which patients can discontinue the IFN-α treatment without imminent disease relapse, yet the patient population studied is small. Prospective clinical trials are needed to confirm its usefulness in decision-making.

Taken together, our results show that IFN-α treatment induces distinct changes in the immunoprofile of CML patients, which may contribute to prolonged therapy responses in this unique group of patients. The potent immunomodulatory effects observed imply that IFN-α still may have a role in the future therapy protocols aiming in permanent cure of CML.

## Supporting Information

Table S1 **Lymphocyte subpopulations analyzed with flow cytometry.** PB, peripheral blood; IFN-ON, CML patients with ongoing IFN-α therapy; IFN-OFF, CML patients who have discontinued the therapy ^a^Continuous variables are expressed as median (minimum-maximum). ^b^Statistical significance of difference is evaluated by non-parametric Kruskal-Wallis test.
(DOC)

Table S2 **TCR γ-gene sequences of clonal lymphocyte populations detected with TCR γ primer pair 5.** The table presents detected TCR γ rearrangements with TCR-γ primer pair 5 (table 3). Sequences of the junction are aligned. No refers to patient number in Table 2.
(DOC)

Table S3 **TCR δ-gene sequences of clonal lymphocyte populations detected with TCR δ primer pair 3.** The table presents detected TCR δ rearrangements. Sequences of the junction region are aligned. No refers to patient number in Table 2.
(DOCX)
