# Supplementary material for: Chronic Myeloid Leukemia Patients in Prolonged Remission following Interferon-α Monotherapy Have Distinct Cytokine and Oligoclonal Lymphocyte Profile
Source: PLoS One. 2011 Aug 9;6(8):e23022. doi: 10.1371/journal.pone.0023022 (PMC3153480; doi:10.1371/journal.pone.0023022)
Supplement: Table S1 — Lymphocyte subpopulations analyzed with flow cytometry. PB, peripheral blood; IFN-ON, CML patients with ongoing IFN-α therapy; IFN-OFF, CML patients who have discontinued the therapy aContinuous variables are expressed as median (minimum-maximum). bStatistical significance of difference is evaluated by non-parametric Kruskal-Wallis test. (DOC) [file pone.0023022.s001.doc]

| **Subpopulation^a^** |  | **Healthy (n = 27)** | **IFN-ON (n = 10)** | **IFN-OFF (n = 9)** | **p-value^b^** |
| --- | --- | --- | --- | --- | --- |
| **CD3+ T-cells** | % of lymphocytes  absolute count in PB (x10^9^/l) | 73 (67-86) 1.3 (0.82-2.18) | 67 (50-78) 0.89 (0.57-1.97) | 56 (30-72) 0.82 (0.45-1.34) | **0.0002 0.0049** |
| **CD4+ cells** | % of T-cells  absolute count in PB (x10^9^/l) | 69 (46-77) 0.84 (0.52-1.65) | 56 (11-82) 0.47 (0.06-1.44) | 45 (12-76) 0.33 (0.17-0.67) | **0.0417 0.0010** |
| **CD8+ cells** | % of T-cells  absolute count in PB (x10^9^/l) | 31 (23-54) 0.44 (0.21-0.82) | 44 (18-89) 0.42 (0.18-0.63) | 55 (24-88) 0.36 (0.21-1.18) | **0.0388** 0.5923 |
| **B-cells** | % of lymphocytes  absolute count in PB (x10^9^/l) | 10 (5-25) 0.19 (0.07-0.58) | 14 (6-20) 0.17 (0.11- 0.32) | 12 (8-28) 0.15 (0.13-0.58) | 0.6114 0.8070 |
| **NK-cells** | % of lymphocytes  absolute count in PB (x10^9^/l) | 11 (5-21) 0.21 (0.09-0.42) | 12 (6-31) 0.19 (0.08-0.53) | 26 (18-51) 0.42 (0.25-0.8) | **0.0005** **0.0012** |
| **NKT-cells** | % of lymphocytes  absolute count in PB (x10^9^/l) | 6 (0.7-15.5) 0.11 (0.01-0.36) | 3 (0.3-7.9) 0.03 (0.01-0.08) | 3 (1.9-4.8) 0.05 (0.02-0.07) | **0.001 < 0.0001** |
| **CD3^+^RA^+^** | % of T-cells  absolute count in PB (x10^9^/l) | 56 (39-70) 0.63 (0.36-1.2) | 34 (15-66) 0.38 (0.14-1.06) | 24 (12-41) 0.23 (0.12-0.87) | **0.0003 0.0005** |
| **CD3^+^RO^+^** | % of T-cells  absolute count in PB (x10^9^/l) | 44 (27-67) 0.6 (0.24-1.01) | 58 (31-85) 0.60 (0.27-1.69) | 74 (55-88) 0.74 (0.47-1.16) | **0.0059** 0.5116 |
| **CD3^+^TCR-αβ^+^** | % of T-cells  absolute count in PB (x10^9^/l) | 95 (85-99) 1.06 (0.4-2.08) | 94 (84-99) 1 (0.52-2.15) | 93 (81-96) 0.89 (0.63-2.86) | 0.5468 0.5190 |
| **CD3^+^TCR-γδ^+^** | % of T-cells  absolute count in PB (x10^9^/l) | 5 (1-14) 0.06 (0.01-0.18) | 4 (1-8) 0.05 (0.01-0.2) | 5 (3-19) 0.06 (0.02-0.15) | 0.3041 0.2935 |
| **CD4^+^TCR-αβ^+^** | % of CD4^+^ T-cells  absolute count in PB (x10^9^/l) | 90 (79-98) 0.4 (0.21-0.83) | 93 (73-98) 0.24 (0.07- 0.59) | 88 (77-95) 0.25 (0.18-0.33) | 0.7159 **0.0037** |
| **CD4^+^TCR-γδ^+^** | % of CD4^+^ T-cells  absolute count in PB (x10^9^/l) | 8 (1-20) 0.04 (0-0.13) | 7 (2-27) 0.02 (0-0.05) | 2 (5-22) 0.04 (0-0.08) | 0.5724 0.0753 |
| **CD8^+^TCR-αβ^+^** | % of CD8^+^ T-cells  absolute count in PB (x10^9^/l) | 97 (93-99) 0.37 (0.19-0.81) | 98 (87-99) 0.43 (0.15-1.19) | 93 (83-98) 0.52 (0.19-2.53) | 0.0985 0.6296 |
| **CD8^+^TCR-γδ^+^** | % of CD8^+^ T-cells  absolute count in PB (x10^9^/l) | 2 (0-6) 0.01 (0-0.04) | 0.5 (0-10) 0.01 (0-0.13) | 4 (0-11) 0.02 (0-0.06) | 0.0783 0.2055 |
| **CD3^+^CD57^+^** | % of T-cells  absolute count in PB (x10^9^/l) | 15 (2-45) 0.16 (0.03-0.8) | 18 (1-44) 0.12 (0.02-0.43) | 12 (8-35) 0.1 (0.07-0.78) | 0.8157 0.7578 |
| **FOXP3^+^ Treg** | % of CD4^+^ T-cells  absolute count in PB (x10^9^/l) | 3.8 (1.4-6.9) 0.03 (0.01-0.06) | 5.2 (2.7-11.9) 0.04 (0.02- 0.1) | 6.1 (2.9-14.1) 0.05 (0.02-0.1) | **0.0114** 0.0672 |

**Table S1**
