# Supplementary material for: Chronic Myeloid Leukemia Patients in Prolonged Remission following Interferon-α Monotherapy Have Distinct Cytokine and Oligoclonal Lymphocyte Profile
Source: PLoS One. 2011 Aug 9;6(8):e23022. doi: 10.1371/journal.pone.0023022 (PMC3153480; doi:10.1371/journal.pone.0023022)
Supplement: Table S2 — TCR γ-gene sequences of clonal lymphocyte populations detected with TCR γ primer pair 5. The table presents detected TCR γ rearrangements with TCR-γ primer pair 5 (table 3). Sequences of the junction are aligned. No refers to patient number in Table 2. (DOC) [file pone.0023022.s002.doc]

**Table S2**

| **Patient** | **Clonal cell population** | **TRGV9*01** | **junction** | **TRGJP*01** | **Function of rearrangement** |
| --- | --- | --- | --- | --- | --- |
| **Germ-line** |  | tgt gcc ttg tgg gag gtg |  | t ggg caa gag ttg ggc aaa aaa atc aag gta ttt |  |
| **1** |  | tgt gcc ttg tgg gag gtg  C A L W E V | gg  # | …..aa gag ttg ggc aaa aaa atc aag gta ttt E L G K K I K V F | Unproductive (out-of-frame junction) |
| **2** |  | tgt gcc ttg tgg gag…  C A L W E | act gg  T G | …….g ttg ggc aaa aaa atc aag gta ttt  L G K K I K V F | Productive |
| **4** | γδ^+^ T-cells | tgt gcc ttg tgg gag gtg  C A L W E V |  | ....caa gag ttg ggc aaa aaa atc aag gta ttt  Q E L G K K I K V F | Productive |
| **5** | γδ^+^ T-cells | tgt gcc ttg tgg gag gt.  C A L W E | c  V | ....caa gag ttg ggc aaa aaa atc aag gta ttt  Q E L G K K I K V F | Productive |
| **6** | γδ^+^ T-cells | tgt gcc ttg tgg gag gtg  C A L W E V |  | ....caa gag ttg ggc aaa aaa atc aag gta ttt  Q E L G K K I K V F | Productive |
| **9** |  | tgt gcc ttg tgg ga....  C A L W E | a atg ggg ttt g  M G F # | .......gag ttg ggc aaa aaa atc aag gta ttt  E L G K K I K V F | Unproductive (out-of-frame junction) |
| **10** |  | tgt gcc ttg tgg gag gt.  C A L W E | c ggc  V G | .......gag ttg ggc aaa aaa atc aag gta ttt  E L G K K I K V F | Productive |
| **11** |  | tgt gcc ttg tgg gag g..  C A L W E | bi-allelic | ……..ag ttg ggc aaa aaa atc aag gta ttt  L G K K I K V F | Unknown |
| **12** |  | tgt gcc ttg tgg gag gtg  C A L W E V | bi-allelic | …….gag ttg ggc aaa aaa atc aag gta ttt  E L G K K I K V F | Unknown |
| **13** | γδ^+^ T-cells | tgt gcc ttg tgg gag gtg  C A L W E V | bi-allelic | bi-allelic | Unknown |
| **14** | γδ^+^ T-cells | tgt gcc ttg tgg gag gtg  C A L W E V |  | ....caa gag ttg ggc aaa aaa atc aag gta ttt  Q E L G K K I K V F | Productive |
| **15** |  | tgt gcc ttg tgg gag gtg  C A L W E V |  | ....caa gag ttg ggc aaa aaa atc aag gta ttt  Q E L G K K I K V F | Productive |
| **17** |  | tgt gcc ttg tgg gag gtg  C A L W E V | c  Q | ........ag ttg ggc aaa aaa atc aag gta ttt  L G K K I K V F | Productive |
| **18** | γδ^+^ T-cells | tgt gcc ttg tgg gag gtg  C A L W E V | ct  L | a gag ttg ggc aaa aaa atc aag gta ttt E L G K K I K V F | Productive |
| **19** |  | tgt gcc ttg tgg gag gtg  C A L W E V |  | ..gg caa gag ttg ggc aaa aaa atc aag gta ttt  # Q E L G K K I K V F | Unproductive (out-of-frame junction) |
