# Supplementary material for: Chronic Myeloid Leukemia Patients in Prolonged Remission following Interferon-α Monotherapy Have Distinct Cytokine and Oligoclonal Lymphocyte Profile
Source: PLoS One. 2011 Aug 9;6(8):e23022. doi: 10.1371/journal.pone.0023022 (PMC3153480; doi:10.1371/journal.pone.0023022)
Supplement: Table S3 — TCR δ-gene sequences of clonal lymphocyte populations detected with TCR δ primer pair 3. The table presents detected TCR δ rearrangements. Sequences of the junction region are aligned. No refers to patient number in Table 2. (DOCX) [file pone.0023022.s003.docx]

| **Patient** | **Clonal cell population** | **TRDV2*03** | **junction** | **TRDD3*01** | **junction** | **TRDJ1*01** | **Function of rearrangement** |
| --- | --- | --- | --- | --- | --- | --- | --- |
| **Germline** |  | tgt gcc tgt gac acc  C A C D T |  | act ggg gga tac g  T G G Y D |  | ac acc gat aaa ctc atc ttt  T D K L I F |  |
| **2** |  | tgt gcc tgt gac  C A C D | ccc ctt t  P L | tg ggg gat |  | ac acc gat aaa ctc atc ttt  # | Unproductive (out-of-frame junction) |
| **4** | γδ^+^ T-cells | tgt gcc tgt gac acc  C A C D T | cg  R | t ggg ggg  G G |  | cg ata aac tca tct tt  # | Unproductive (out-of-frame junction) |
| **5** | γδ^+^ T-cells |  |  |  |  |  |  |
| **8** |  | tgt gcc tgt gac acc  C A C D T |  |  |  | ac acc gat aaa ctc atc ttt  # | Unproductive (out-of-frame junction) |
| **9** |  | tgt gcc tgt gac acc  C A C D T | ggg t  G | ac tgg ggg  T W G | cga ccg t  R P | ac acc gat aaa ctc atc ttt  Y T D K L I F | Productive |
| **11** | γδ^+^ T-cells | tgt gcc tgt gac  C A C D | tca gt  S V | a ctg g  L | ta tc  V S | c acc gat aaa ctc atc ttt  T D K L I F | Productive |
| **12** | γδ^+^ T-cells | tgt gca tgt gac  C A C D | acc gca ag  T A | a ctg ggg gat acg  # | gtg ggt ggg ttt aac atc cca gag  # | taa act cat ctt t  # | Unproductive (out-of-frame junction) |
| **13** |  | tgt gcc tgt gac  C A C D | xxx | xxx | xxx | cc gat aaa ctc atc ttt | Unknown (junction region bi-allelic) |
| **14** |  | tgt gcc tgt  C A C |  |  |  | aaa ctc atc ttt  K L I F | Productive |
| **15** |  | tgt gcc tgt gac  C A C D | cct ttg cct tct gag gga ggt ata gc  P L P S E G G R A | t ggg gga tat  G G Y | ctc cct tac ta  L P Y Y | c gat aaa ctc atc ttt  D K L I F | Productive |
| **19** |  | tgt gcc tgt gac acc  C A C D T | cat cg  H R |  |  | c gat aaa ctc atc ttt  D K L I F | Productive |

**Table S3**
